# Vaccines for maternal immunization against Group B Streptococcus disease: WHO perspectives on case ascertainment and case definitions

**DOI:** 10.1016/j.vaccine.2019.07.012

**Published:** 2019-08-14

**Authors:** Anna C. Seale, Carol J. Baker, James A. Berkley, Shabir A. Madhi, Jaume Ordi, Samir K. Saha, Stephanie J. Schrag, Ajoke Sobanjo-ter Meulen, Johan Vekemans

**Affiliations:** a London School of Hygiene & Tropical Medicine, Keppel Street, London, UK; b College of Health and Medical Sciences, Haramaya University, Harar, Ethiopia; c KEMRI-Wellcome Trust Research Programme, Kilifi, Kenya; d Department of Pediatric, University of Texas Health Science Center McGovern Medical School, Houston, TX, USA; e Centre for Tropical Medicine, University of Oxford, Oxford, UK; f Medical Research Council: Respiratory and Meningeal Pathogens Research Unit, & Department of Science and Technology/National Research Foundation: Vaccine Preventable Diseases, University of the Witwatersrand, Faculty of Health Sciences, Johannesburg, South Africa; g ISGlobal, Barcelona Institute of Global Health, Barcelona, Spain; h Department of Pathology, Hospital Clinic of Barcelona, Universitat de Barcelona, Barcelona, Spain; i Bangladesh Institute of Child Health, Dhaka, Bangladesh; j National Center for Immunization and Respiratory Diseases, Centers for Disease Control and Prevention, Atlanta, GA, USA; k Bill & Melinda Gates Foundation, Seattle, USA; l World Health Organization, Geneva, Switzerland

**Keywords:** CIDT, culture independent diagnostic test, **EOGBS**, early onset GBS disease, GBS, Group B *Streptococcus*, LOGBS, late onset GBS disease, LMICs, low and middle income countries, NDI, neurodevelopmental impairment, UR, uncertainty range, WGS, whole genome sequence, WHO, World Health Organization, Streptococcus agalactiae, Group B, Case definition, Case ascertainment, Vaccine

## Abstract

Group B *Streptococcus* (GBS) is an important cause of disease in young infants, stillbirths, pregnant and post-partum women. GBS vaccines for maternal immunization are in development aiming to reduce this burden. Standardisation of case definitions and ascertainment methodologies for GBS disease is needed to support future trials of maternal GBS vaccines. Considerations presented here may also serve to promote consistency in observational studies and surveillance, to better establish disease burden. The World Health Organization convened a working group to provide consensus guidance for case ascertainment and case definitions of GBS disease in stillbirths, infants, pregnant and post-partum women, with feedback sought from external stakeholders. In intervention studies, case capture and case ascertainment for GBS disease should be based on antenatal recruitment of women, with active follow-up, systematic clinical assessment, standardised sampling strategies and optimised laboratory methods. Confirmed cases of invasive GBS disease in stillbirths or infants should be included in a primary composite endpoint for vaccine efficacy studies, with GBS cultured from a usually sterile body site (may be post-mortem). For additional endpoints, or observational studies, confirmed cases of GBS sepsis in pregnant and post-partum women should be assessed. Culture independent diagnostic tests (CIDTs) may detect additional presumed cases, however, the use of these diagnostics needs further evaluation. Efficacy of vaccination against maternal and neonatal GBS colonisation, and maternal GBS urinary tract infection could be included as additional, separate, endpoints and/or in observational studies. Whilst the focus here is on specific GBS disease outcomes, intervention studies also present an opportunity to establish the contribution of GBS across adverse perinatal outcomes, including all-cause stillbirth, preterm birth and neonatal encephalopathy.

## 1 Introduction

### 1.1 Burden of disease and interventions

Group B *Streptococcus* (GBS) causes invasive disease in pregnant and post-partum women, infants and fetuses, resulting in maternal and infant disease, death or disability, and stillbirth. Recent annual estimates of 319,000 (uncertainty range (UR) = 119,000–417,000) infant GBS cases and 90,000 (UR = 41,000–185,000) infant GBS deaths worldwide [1] are higher than those for other diseases for which maternal vaccines are recommended or are further along in development, such as influenza and respiratory syncytial virus. In addition, although data are limited, it is conservatively estimated that 57,000 (UR 12,000–104,000) stillbirths are associated with GBS, and 33,000 (UR = 13,000–52,000) pregnant or puerperal women have GBS sepsis each year [1]. For those infants that survive GBS disease, there can be long term neurodevelopmental impairment (NDI) [2], however, data are currently insufficient to estimate this burden beyond NDI associated with GBS meningitis. Maternal GBS colonisation can also be associated with preterm birth, but data are also insufficient to quantify this [3].

The reservoir for GBS in humans is the gastrointestinal tract and maternal recto-vaginal colonisation is necessary for ascending fetal infection and stillbirth/early onset GBS disease (EOGBS, days 0–6 after birth). In some high- and middle-income settings, maternal recto-vaginal colonisation has been used to guide intrapartum antibiotic prophylaxis (IAP) to prevent early-onset GBS disease [4], [5], based on either microbiological detection of GBS or the presence of clinical risk-factors. However, whilst reductions in EOGBS in the USA have been observed [6], IAP does not prevent late onset GBS disease (LOGBS, days 7–89) [7] and is unlikely, given the timing of prophylaxis, to impact on stillbirth or preterm birth. The development of GBS vaccines suitable for maternal immunization in pregnancy and use in low- and middle-income countries (LMICs) has been identified as a priority by the World Health Organization (WHO). Key consensus documents, highlighting preferences for GBS vaccine product characteristics and providing a research and development technical roadmap, have been developed to accelerate vaccine availability [8], [9], [10].

Recent Brighton Collaboration guidelines have been developed for defining neonatal infection [11], but strengthening of case capture methodologies and definitions of specific clinical endpoints for GBS have been identified as a critical need for robust estimates of effect in maternal GBS vaccine trials and studies of immune correlates of protection [10]. In addition, considerations presented here support collection of standardised data and sampling for GBS disease in pregnant women, stillbirths and infants, to allow meaningful comparisons across studies. More data on GBS disease incidence are needed across geographical settings, particularly Asia, but also sub-Saharan Africa, and in particular, West Africa [1].

### 1.2 Prioritization of clinical vaccine efficacy endpoints

Two main licensure pathways are being considered for maternal GBS vaccines. One is demonstration of vaccine efficacy against specific clinical endpoints, in randomized controlled trials. The other is registration on the basis of vaccine immunogenicity with immune correlates of protection against specific clinical disease endpoints, established through seroepidemiological studies [9]. The former would provide gold standard evidence of protection and an estimate of the overall public health impact of vaccination, but would require a large and costly study, difficult to conduct in the context of access to high standards of care [12]. A licensure pathway based on immune correlates of protection may lead to faster product availability, at reduced cost, without the need for a very large pre-licensure trial. However, post licensure, introduction probe studies would still offer an opportunity to define public health impact. For both validation of immunological correlates of protection and for a vaccine efficacy trial, standard case definitions and ascertainment methodologies are needed for specific clinical disease endpoints. Guidance on immune correlates is beyond the scope of this paper, and considered elsewhere [13].

Culture confirmed invasive GBS disease in young infants would likely meet regulatory requirements as a highly specific primary endpoint, with public health relevance. However, the relatively low incidence of invasive infant GBS disease reported (in observational studies) means that this would require very large (approximately 70,000 pregnant women) clinical efficacy trials [12]. Invasive infant GBS disease is, however, likely substantially under-reported in data from observational studies. The reported incidence of any infant infectious disease depends on how the population denominator is defined, whether potential cases access health care (usually either through parental concern or referral by community health workers in low-income settings), how cases are clinically assessed, how cases are selected for biological sampling, and how samples are taken and processed for a specific diagnosis [14]. Variations in any of these factors affect case capture and ascertainment, and bias estimates of disease incidence. The risk of low case capture and under-ascertainment are highest in observational studies and surveillance in LMIC settings where access to health care is usually more limited (care-seeking for neonates ranges from 10 to 100%) [15], clinical staff are scarcer [16], sample-taking is restricted by resources, and availability of quality controlled laboratories to detect infection with appropriate methods is limited. For invasive infant GBS disease, low case capture is likely to be particularly high, as many cases occur at, or within a few hours, of birth, and there is rapid disease progression and a high case fatality risk (e.g., 61% in the first 24 h after delivery for neonatal GBS cases in a hospital in Kenya) [17]. Increasing case capture and ascertainment of invasive infant GBS disease reduces sample size requirements, but requires optimisation of methods for case capture, clinical sampling and laboratory detection.

Invasive infant GBS disease is most common in the first few days of life, and arises from ascending GBS infection from the maternal genito-urinary tract. Fetal infection *in utero* presents with clinical signs at, or shortly after, delivery. However, fetal demise may also occur *in utero*, and the baby is stillborn as a result of GBS infection. Recent work suggests 1–4% of all stillbirths (born with no signs of life and ≥28 weeks’ gestation, or >1000 g birth weight) are associated with GBS [18], [19], with recent studies in Kenya [17], Mozambique [20] and South Africa (personal communication, Madhi, S.). Sampling post-mortem of sterile sites offers the opportunity to detect GBS-associated stillbirth. Furthermore, the same techniques can be used to detect GBS disease in neonates and infants who have died before reaching care and/or investigation for infection, which can be used to increase detection of invasive GBS disease [21]. A primary composite endpoint of serious and fatal events associated with GBS, including cases of invasive GBS disease in neonates, infants and stillbirths would be highly relevant as a common entity, better reflect true burden and reduce sample size requirements for estimation of the effect of new interventions.

Additional endpoints for consideration include invasive maternal GBS disease, as well as maternal and neonatal GBS colonisation, and maternal GBS urinary tract infection. Maternal sepsis is a leading cause of maternal death, with much higher incidence in South Asia and sub-Saharan Africa than in higher-income regions. Studies in these settings show sepsis to account for 14% (95% confidence interval [CI] 3%–36%) and 10% (95%CI 5.5, 18.5) of all maternal deaths respectively [22], compared to 4.7% (95%CI 2⋅4, 11⋅1) in high-income countries [22]. Worldwide, data on the aetiology of maternal sepsis (often treated empirically) are limited, but GBS has been shown to be a frequent contributor to maternal sepsis in high-income countries [23], [24], [25], [26], [27]. It accounted for 25% of maternal bacteraemia in Ireland [28] and 20% of puerperal bacteraemia in the USA before implementation of screening and treatment guidelines [27]. Data on the incidence of GBS sepsis in pregnant and postpartum women in LMICs are lacking, but it may be an important contributor to maternal sepsis [29]. In contrast, maternal GBS colonisation is known to be common, with 10–40% of pregnant women colonised with GBS [30], [31]. Of these, around 30–50% will have newborns where GBS is detected shortly after birth [17]. In the absence of intervention 1.1% (95% CI 0.6–1.5%) of newborns born to women colonised with GBS will have EOGBS, decreasing to 0.3% (0–0.9%) with high (80%) coverage of microbiological screening during late pregnancy and administration of intrapartum antibiotic prophylaxis [32]. The impact of vaccination on maternal GBS colonisation will be important to characterize in terms of risk of disease through exposure [33], and changes in circulating serotypes, through serotype-specific reduction. Culture-confirmed maternal GBS urinary tract infection could also be included as an endpoint, particularly as it can precede invasive GBS disease in mothers or the fetus. Additional clinical samples for diagnosis of maternal chorioamnionitis, postpartum endometritis, and mastitis may be of interest.

Here we suggest case capture and case ascertainment methodologies and definitions, prioritizing case ascertainment and case definitions for the most specific and serious infections in the fetus, neonate and young infant, aiming to provide a simple and pragmatic approach applicable across settings (summarised in Table 1). We also include invasive GBS disease in pregnant and postpartum women, and specific details pertaining to maternal and neonatal GBS colonisation (Panel 1) and maternal urinary tract infection. However, intervention studies also provide an important opportunity for vaccine probe-studies to investigate other adverse perinatal outcomes associated with maternal GBS colonisation and ascending infection (with or without invasive disease), including preterm birth and neonatal encephalopathy [3], [34]. The recommendations presented here are based on definitions identified in systematic literature reviews to estimate the burden of GBS disease [29], [30], [32], [35], [36], [37], [38], [39], [40], [41], discussion with an expert group convened by the World Health Organization, and consideration of endpoints used for other vaccine preventable childhood infections. The expert working group reviewed drafts of case ascertainment methodologies and case definitions and we shared these with external stakeholders for feedback.

**Table 1:** Study methodologies and case ascertainment for invasive Group B Streptococcus (GBS) disease in pregnant, post-abortion and post-partum women, stillbirths and young infants (0–89 days) for observational and clinical vaccine efficacy trials.

	Observational epidemiological studies or surveillance	Clinical vaccine efficacy trials
Study design	Cohort studies with antenatal recruitment, and follow-up through 89 days after delivery, are preferred. Surveillance can be used to provide incidence data with denominators based on hospital catchment population, or facility based births. However, both are subject to bias which should be assessed and reported.	Randomised controlled trial. Follow-up for efficacy at least 89 days after delivery.
Study population for case capture	Inclusion of stillbirths and infants (0–89 days) preferred. Consider inclusion of pregnant, post-abortion and post-partum women.	Inclusion of stillbirths, infants (0–89 days) recommended for primary composite endpoint. Consider inclusion of pregnant, post-abortion and post-partum women for additional endpoints.
Clinical case definition	Standardised clinical assessment using defined clinical criteria preferred. Local adaptations should be clearly reported.	Standardised clinical assessment and sampling based on presence of defined clinical criteria essential.
Sampling	Sampling based on presence or absence of clinical signs; samples collected with aseptic technique, at recommended volumes according to age/weight. Post-mortem sampling recommended.	Sampling based on presence or absence of clinical signs; samples collected with aseptic technique, at recommended volumes according to age/weight. Post-mortem sampling recommended.
Laboratory characterization	Standardised operating procedures with sensitive methods (automated blood cultures, selective broth and agar). Inclusion of culture independent diagnostic tests where feasible. All isolates stored, serotype and/or whole genome sequencing where feasible.*	Standardised operating procedures with sensitive methods (automated blood cultures, selective agar), and quality assurance according to clinical development stage. Inclusion of culture independent diagnostic tests as secondary endpoint. All isolates stored, whole genome sequencing of isolates. *

* All GBS isolates should be typed or stored for later typing, ideally using whole genome sequencing*.*

Panel 1 **Ascertainment of maternal and neonatal GBS colonisation.**

*Why investigate colonisation?*

● Maternal GBS colonisation prevalence is important, to assess the risk of GBS infection to the fetus/newborn, and any increased risk of preterm birth and/or neonatal encephalopathy from ascending (but non-invasive) GBS [34].

● Determination of neonatal GBS contamination at delivery, or subsequent colonisation (>24 h after birth) can be important to answer additional questions relating to GBS transmission, and risk of late-onset GBS disease, respectively.

● Colonizing GBS isolates which are typed increase the data available on circulating strains and serotypes, and can be used before and after vaccine introduction to monitor serotype replacement and/or capsular switching.

*Sampling*

● For pregnant women, sampling at delivery (in stage 1 or 2 of labour) is recommended, including women of all gestations. This will provide data to test the association of maternal GBS colonisation at delivery with preterm birth. As maternal GBS colonisation can be intermittent, sampling at delivery is considered most relevant to generate inferences about GBS impact on pregnancy outcomes, even though peri-partum antibiotic exposure, or prolonged rupture of membranes (≥18 h) and other obstetric complications (e.g., antepartum hemorrhage) [17], may reduce GBS detection. The presence of these factors should be documented. Earlier sampling may, in addition, be useful to study GBS acquisition.

● Site of sampling (applicable in pregnant or non-pregnant women) for maximum sensitivity this should include both low vaginal and rectal sampling (wiping the swab in a circle around the mucosa of the lower 1/3 of the vagina and in a circle around the mucosa of the lower rectum, through the anal sphincter) [64], [65], [66], [67]. Dependent on the study question it may be preferable to have two swabs, rather than one combined (as usually done for the purposes of determining intrapartum antibiotic prophylaxis), but in all cases should allow maternal GBS colonisation prevalence to be calculated based on combined low vaginal and rectal sampling to maximize sensitivity.

● For newborns the time of sampling depends on whether GBS exposure of the baby at delivery is of interest (may indicate risk of EOGBS). In this case, sampling <48 h of delivery, ideally <6 h of delivery would be appropriate and sample sites would include ear, umbilicus, and nares, with isolation of GBS from any one indicating exposure. Evidence of vertical transmission can be supported with paired maternal-newborn isolates and whole genome sequencing. Sampling >24–48 h is more likely to indicate neonatal GBS colonisation which may be of interest in terms of assessing risk of GBS exposure at delivery resulting in colonisation, and/or risk of late-onset invasive disease, as well as detecting horizontal GBS acquisition. Colonisation would be expected at areas of mucosal replication, so throat and rectal sites (through anal sphincter) should be swabbed, separately.

*Laboratory methods*

● Colonisation is tested with swabs which should be stored in a non-nutritive transport medium (such as Amies or Stuart with or without charcoal) prior to processing, ideally within 24 h. To reduce potential loss of viability, if transported, samples should be kept in cool storage (4 °C) then processed <48 h [68].

● Antibiotic broth enrichment (such as LIM; Todd-Hewitt broth with colistin and nalidixic acid or TransVag; Todd-Hewitt broth with gentamicin and nalidixic acid) should be used for 24 h prior to subculture. Subculture onto an appropriate agar plate (such as 5% sheep blood agar, neomycin-nalidixic acid (NNA) agar, Columbia colistin and naladixic acid (CNA) agar with 5% sheep blood, chromogenic agar or other selective *Streptococcus* agar.). Chromogenic pigments in broth or media can be used but a negative result does not exclude GBS, as isolates may not be B-haemolytic.

● GBS isolation supports storage and whole genome sequencing of isolates and is thus currently preferable to DNA detection.

## 2 Study methodologies and case ascertainment

### 2.1 Study design

**Fig. 1:**
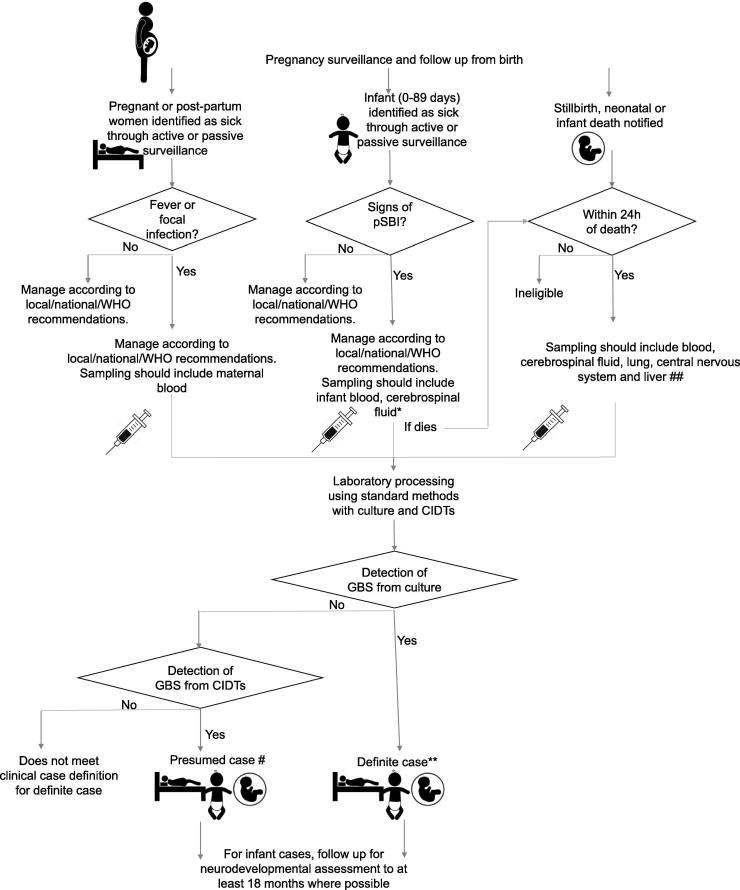
Case ascertainment of invasive GBS disease in pregnant and post-partum women, stillbirths and infants (0**–**89 days). pSBI = possible serious bacterial infection; CIDT = culture independent diagnostic tests. *Unless clinical contraindication. **GBS isolates should be stored, to allow later typing, ideally using whole genome sequencing. # Dependent on establishment of specificity of the particular CIDT (based on nuclei acid amplification) used. ## Blood, cerebrospinal fluid and lung should be prioritised for sampling.

A randomized controlled trial (RCT) with the most relevant clinical endpoint would constitute the gold standard design for estimation of vaccine efficacy (Table 1, Fig. 1), but the challenges for maternal GBS vaccine trials are acknowledged. Observational studies and surveillance should ideally be conducted at the same level of rigour as RCTs. For observational studies, therefore, case capture would ideally be through recruitment of a cohort of pregnant women, but without the intervention. However, this may not always be feasible, and the use of population catchment or facility birth denominators are pragmatic in surveillance, and can support estimation of incidence of EOGBS and late onset GBS disease (LOGBS). Where these strategies are used, assessment and/or mitigation of limitations should be considered. For studies using population catchment estimates, selection bias can be approximated through a health utilization survey. For studies in health facilities and/or in the community, under-ascertainment can be reduced by investigation of infant deaths, particularly in those who do not reach care. Where facility births are used as a denominator, it is important to consider and report that the disease incidence observed may not reflect the true population incidence, and/or case fatality risk, as women delivering in the facilities are unlikely to be representative of the general population in some settings, and case fatality risks are likely to be lower, particularly in LMICs.

### 2.2 Study population for case capture

For vaccine efficacy studies (Table 1) it is essential to recruit women during pregnancy, according to defined eligibility criteria, randomise to receive the intervention or not, and follow up mothers and newborns for at least 90 days after delivery. The primary composite endpoint would include stillbirths and infants (0–89 days) (Fig. 1). Characterization of post-abortion and post-partum maternal outcomes would also be relevant. Inclusion of all these outcomes would be preferable in observational studies too. Case capture can be increased in all study designs with a defined schedule of follow-up visits after birth, and, most importantly (but most challenging) including the day of birth, when most early deaths occur [42] and the majority (75–90%) of neonates with early onset GBS (EOGBS) disease present [43]. Follow-up of infants with invasive GBS disease (and appropriate controls) for at least 18 months is needed if neurodevelopmental impairment outcomes are to be included, and this should be considered to better understand the burden of long-term neurodevelopmental impairment associated with invasive GBS disease in infancy.

### 2.3 Clinical characterisation – Neonates and infants

Systematic clinical assessment and sampling methods should be implemented for observational studies and surveillance, as for randomised controlled trials. Clinical algorithms are used to guide empirical treatment for infants with possible serious bacterial infection (pSBI), based on the presence of pre-specified clinical signs (Table 2) [44], [45]. Current guidelines suggest infants with pSBI are referred to hospital [46], although outpatient treatment may be offered where referral is not possible [47]. Using these guidelines, health care workers should document the presence or absence of specific clinical signs in accordance with international guidelines, with flexibility to include additional documented signs in accordance with local protocols. These should be used to guide systematic clinical sampling, using an aseptic technique to sample from sterile sites (blood and CSF). Sampling should be prior to antibiotic administration, as long as this does not delay empirical treatment. Antibiotic administration prior to sampling (e.g., when given prior to hospital referral), contamination, and low blood volumes decrease sensitivity of GBS detection using conventional culture methods.

**Table 2:** Case definitions for invasive Group B Streptococcus (GBS) disease in pregnant, post-abortion and post-partum women, stillbirths, neonates and young infants (0–89 days).

Case criteria	Clinical criteria	Laboratory criteria for confirmed GBS disease*	Laboratory criteria for presumed GBS disease (secondary case definitions)
Stillbirth (born with no signs of life and ≥28 weeks’ gestation or >1000 g body weight)	Foetal demise.	Isolation of GBS from blood, CSF, lung, CNS or liver.	Culture independent diagnostic tests detect GBS from blood, CSF, lung, CNS or liver.
Neonates and young infants (through 89 days)	Infant death and/or ≥1 clinical signs of possible serious bacterial infection: • Temperature ≥37.5 ◦C or <35.5 °C • Tachypnoea (≥60 breaths per minute) or severe chest indrawing or grunting or cyanosis • Change in level of activity • History of feeding difficulty • History of convulsions.	Isolation of GBS from blood or cerebrospinal fluid in live infants and/or isolation of GBS from blood, CSF, lung, CNS or liver in infants who have died.	Culture independent diagnostic tests detect GBS from blood or cerebrospinal fluid in live infants, and/or from blood, CSF, lung, CNS or liver in stillbirths or infants who have died.
Pregnant, post-abortion and postpartum women up to 42 days post delivery	Fever >38 °C or clinical suspicion of sepsis, or history of fever and signs of endometritis (abdominal pain, or foul smelling vaginal discharge) or chorioamniontis.	GBS isolated from maternal blood.	GBS identified from culture independent diagnostic tests from maternal blood.

* All GBS isolates should be typed or stored for later typing, ideally using whole genome sequencing.

Experience investigating neonatal deaths for GBS disease is currently limited. The CaDMIA and CaDMIA plus studies (Cause of Death using Minimally Invasive Autopsies) have recently provided data comparing minimally invasive tissue sampling (MITS, also termed minimally invasive autopsy (MIA)), with complete diagnostic autopsy (CDA) in Mozambique. In neonates, only three GBS infections were detected by CDA, and none by MITS [20]. In stillbirths, three cases of GBS infection were identified by CDA and the same three by MITS. Further data are awaited from ongoing studies in seven sites across South Asia and sub-Saharan Africa as part of the Child Health and Mortality Prevention Surveillance (CHAMPS) network [48]. The CHAMPS protocol suggests including 1.5 mL blood (sampled from the subclavian vein or the heart), liver tissue (12 specimens), lung tissue samples (six chest punctures (both left and right superior, mid and inferior chest wall), with four tissue specimens from each entry point), CSF sampling and central nervous system tissue samples (12 specimens obtained through occipital, trans-nasal puncture and/or through the anterior fontanelle). Where it is not acceptable, practical or feasible to undertake sampling to this extent, for consistency with investigation of sick neonates, we suggest blood and CSF sampling should be prioritised, as well as the lung, where GBS is sequestered [49].

There is currently no established gold standard for the timing of samples, but this should be as soon as possible after death, to reduce the chance of post-mortem bacterial overgrowth resulting in false positives. We suggest sampling within 24 h of death, in line with the current CaDMIA plus and CHAMPS programmes. Suggested timing and sample taking may need to be revised when new data from CaDMIA plus and CHAMPS are available. Surface GBS colonisation should not be considered sufficient to attribute death to GBS disease.

### 2.4 Clinical characterisation – Stillbirths

There are limited data on case ascertainment of GBS associated stillbirth, with the most recent studies from Kenya [17], Mozambique [20], and South Africa (in progress). As GBS has been identified in both antepartum and intrapartum stillbirths, all stillbirths meeting the WHO definition for stillbirth (born with no signs of life ≥28 weeks’ gestation or >1000 g) should be included in sampling strategies for consistency across settings.

Sampling methods, to date, have been varied, including blood (cord or heart), lung needle aspirate, minimally invasive autopsy (blood, CSF and multiple organ sampling), and CDA [20]. Again, more data are expected from CHAMPS and CaDMIA plus [48]. In the interim, the CHAMPS protocol recommends samples of blood (minimum 1.5 mL sampled from the subclavian vein or the heart), lung, liver, central nervous system and CSF are taken as possible after death, but within 24 h after delivery. As for neonates, where sampling to this extent this is not acceptable, practical or feasible, we suggest prioritizing blood, cerebrospinal fluid and lung.

### 2.5 Clinical characterisation – Pregnant, post-abortion and post-partum women up to 42 days post delivery

A new WHO consensus definition for maternal sepsis has recently been defined as a “life-threatening condition with organ dysfunction resulting from infection during pregnancy, child-birth, post-abortion, or postpartum period” [50]. Clinical criteria are in the process of being validated for this diagnosis, and as a first step will include identification of women with possible severe maternal infection [50]. The diagnostic criteria for possible severe maternal infection may serve as appropriate, sensitive clinical criteria for sampling pregnant, post-abortion, and postpartum women for maternal sepsis [50]. However, until standard clinical criteria are determined, a simple, sensitive and pragmatic approach is needed to guide case ascertainment for maternal GBS sepsis. We suggest that all pregnant, post-abortion and postpartum women up to 42 days after delivery with a temperature >38 °C should be investigated with blood sampling (for culture and culture-independent diagnostic tests (CIDTs)), or where sepsis is clinically suspected, or where there is a history of fever and clinical suspicion of endometritis (abdominal pain or foul smelling vaginal discharge) or chorioamnionitis [51]. This is suggested as a sensitive approach, to maximise case detection. Specificity is provided through microbiological testing rather than clinical case definition only.

Maternal urinary tract infection can include asymptomatic bacteriuria, acute cystitis and pyelonephritis. Asymptomatic bacteriuria would only be detected through routine screening, but clinical symptoms of acute cystitis (frequency, urgency, dysuria) or pyelonephritis (fever, and flank pain, or nausea or vomiting, which may be associated with symptoms of acute cystitis) can direct investigation for urinary tract infection, to include a mid-stream urine sample.

### 2.6 Laboratory characterisation – Detection

Microbiological methods should be sensitive and specific and at present conventional cultures are the gold-standard, and are preferred to confirm cases as they have high specificity. CIDTs using nucleic acid amplification methods may increase case ascertainment, but this may be at the cost of specificity. This should be assessed in existing CIDTs and those developed in future, including the use of controls in observational studies [52]. Culture and isolation of GBS also has the advantage that isolates can be assessed for antibiotic susceptibility using standard methods and up-to-date thresholds [53], [54], [55], and serotype based on capsular polysaccharide, or using ST typing [56] and/or whole genome sequencing to determine phylogeny and clonal complex type [57], [58]. Sensitivity of conventional microbiological culture methods should be optimised through collection of appropriate sample volumes (based on age and weight) prior to antibiotic administration, and automation of blood cultures to support standardisation.

### 2.7 Laboratory characterisation – Typing

Serotype identification for GBS was originally through capillary precipitation (serotypes Ia, Ib, II, and III) [59], but latex agglutination assays are now commercially available for 10 serotypes Ia/Ib/II-X [60], [61]. PCR and whole genome sequencing also allow serotype assignment according to the genes present [62], [63]. Although whole genome sequencing does not provide information on gene expression (which could become important if more GBS disease is caused by serologically non-typeable GBS), it does enable detailed phylogenetic examination of multiple pathogen genomes, their evolution, assessment of transmission, virulence factors and assessment of any polymorphisms in the protein antigen sequence (for protein vaccines), as well as the relationship between serotype, antimicrobial susceptibility^31^ and GBS clonal complex (CC-1, CC-10, CC-19, CC-17 and CC-23), to be investigated. This latter point will be particularly important if serotype-specific vaccines are introduced, and there is capsular switching in a virulent clone, such as clonal complex 17, currently almost exclusively serotype III. It is also important in order to detect protein antigen target polymorphism if protein vaccines are trialled. GBS isolates should be stored for future characterization if whole genome sequencing is not readily available.

## 3 Case definitions for invasive disease

### 3.1 Neonatal and young infant GBS disease

To confirm a case of GBS invasive disease (sepsis or meningitis), there should be microbiological confirmation of GBS isolation from a sterile site in neonates/young infants with ≥1 clinical sign of possible serious bacterial infection, or death (Table 2). Sampling sites to meet this definition should be blood or cerebrospinal fluid in live infants, and blood, cerebrospinal fluid, central nervous system, liver or lung in infants who have died (Table 2). This is consistent with recent Brighton consensus guidelines on the diagnosis of neonatal sepsis of all aetiologies, at Level 1 (best evidence) [11]. Presumed cases rely on the same demographic and clinical criteria, but GBS is detected by culture independent diagnostic tests (CIDT) from sterile sites as above, with defined specificity and sensitivity.

### 3.2 GBS-associated stillbirth

To confirm a case of GBS associated stillbirth (fetal disease), a consistent approach with neonatal/infant disease should be taken, with cases confirmed by post-mortem microbiological isolation of GBS from a sterile body site (blood, cerebrospinal fluid, central nervous system, lung or liver). Presumed cases are where GBS is detected by CIDTs from usually sterile sites, with defined specificity and sensitivity. Isolation/detection of GBS from a surface or placenta swab only would not be included as a case. Further research is encouraged to define the role of histological evidence of chorioamnionitis in the presence of a GBS positive placental surface swab in case ascertainment.

### 3.3 Maternal GBS sepsis and urinary tract infection

To confirm a case of maternal GBS sepsis, there should be microbiological isolation of GBS from blood of pregnant, post-abortion and postpartum women up to 42 days after delivery with fever (>38 °C) or history of fever and clinical suspicion of endometritis or chorioamnionitis. Presumed cases are where GBS is detected by CIDTs from usually sterile sites, with defined specificity and sensitivity. For confirmation of urinary tract infection, there should be microbiological isolation of >10^5^ GBS colony forming unit/mL from a mid-stream urine sample in symptomatic women.

## 4 Conclusions

Our proposed case definitions and methods for case capture and case ascertainment focus on confirmed invasive GBS disease in stillbirths and infants, providing a framework for use in vaccine efficacy trials, observational studies and surveillance. Key considerations on additional endpoints including maternal outcomes are also presented. The contribution of GBS to non-culture confirmed stillbirth and infant disease, as well as of GBS to preterm birth and neonatal encephalopathy, may in the future be investigated in vaccine probe studies.

High standard case capture and case characterization will likely require capacity strengthening into infrastructures and know-how, in resource-limited settings. Improved approaches to investigation of infant deaths, particularly in the community, are required, and the subject of current studies. The evidence provided will ultimately support optimal policy decisions and appropriate use of potentially life-saving new interventions.

## Contributors

JV had the rationale for this work and convened the international WHO expert group. ACS wrote the initial draft, and oversaw the manuscript revisions. CJB, JAB, SAM, JO, SKS, SJS, AStM, JV contributed to discussions, and development and revisions of the manuscript. All authors agreed to the final draft of the manuscript.

## Disclosures

CJB has undertaken consultancy work for Pfizer Inc. SAM has collaborated on GBS grants funded by Glaxo Smith Kline and by Pfizer Inc. and received personal fees for being member of its advisory committee; he has also collaborated on a GBS grant funded by BMGF with Minervax. AStM works for the Bill & Melinda Gates Foundation, and contributed to discussions, and development and revisions of the manuscript.

## Sources of support

ACS is funded by The Wellcome Trust (205184). This work was supported by a grant from the Bill & Melinda Gates Foundation to WHO (OPP1134011). The corresponding author had access to all data in the study and had responsibility for the decision to submit for publication.

## Declaration of Competing Interest

The authors declare no competing financial interests.
